# Green Spaces and Health Outcomes in Older Adults: A Bibliometric Analysis

**DOI:** 10.1155/jare/6598569

**Published:** 2025-12-08

**Authors:** Ayşe Seval Palteki

**Affiliations:** ^1^ School of Public Health and Emergency Management, Southern University of Science and Technology, Shenzhen, Guangdong, China, sustc.edu.cn; ^2^ School of Medicine, Istanbul Medipol University, Istanbul, Türkiye, medipol.edu.tr

**Keywords:** aging in place, green spaces, older adults, urban planning

## Abstract

**Background:**

As global populations age, promoting the health and well‐being of older adults has become a public health priority. Green spaces—such as parks, gardens, and forests—offer numerous health benefits, including enhanced physical activity, mental well‐being, and social interaction. This study employs a bibliometric analysis to explore global research trends on the relationship between green spaces and older adults’ health.

**Methods:**

Data were collected from the Web of Science Core Collection (WoSCC) and Scopus databases, covering the period from 2004 to 2025. A total of 2066 relevant studies were identified and analyzed in terms of publication trends, geographical contributions, key research themes, and influential works.

**Results:**

Results indicate a growing body of research, with the United States, China, and Australia emerging as leading contributors. Keyword clustering highlights major research themes, including mental health, built environment, aging in place, and urban planning. Despite increasing recognition of the importance of green spaces for older adults, gaps remain in equitable access, longitudinal studies, and intervention‐based research.

**Conclusion:**

The findings emphasize the need for policy‐driven urban planning that not only prioritizes green space accessibility but also addresses equity and supports healthy aging.

## 1. Introduction

As the global population ages, addressing the health and well‐being of older adults has become a public health priority. Older adults are particularly vulnerable to noncommunicable diseases, physical decline, and social isolation, which significantly affect their quality of life. In this context, green spaces—defined as outdoor environments with natural elements such as parks, gardens, and forests—offer an underexplored yet promising avenue for promoting health in older populations.

Green spaces provide opportunities for physical activity, social interaction, and exposure to nature, all of which contribute to overall well‐being. Recent studies have highlighted the positive impact of green spaces on reducing stress levels, improving cardiovascular health, and fostering cognitive functioning among older adults [1–3]. Furthermore, access to green environments may help mitigate feelings of loneliness and social isolation, which are prevalent issues in older populations [4].

The global trend of population aging underscores the growing relevance of understanding green spaces’ influence on the health of older adults. Although bibliometric and systematic reviews have proven valuable for mapping this field, existing studies have predominantly concentrated on mental health and quality‐of‐life outcomes [2, 5]. In contrast, the present research employs bibliometric analysis to systematically investigate a more comprehensive spectrum of health effects specifically within the older demographic, thereby addressing a notable gap in the current body of literature.

This study aims to explore global research trends on green spaces and older adults through bibliometric analysis, focusing on publication outputs, influential countries, institutions, authors, and collaborations. The insights derived from this analysis could inform future research and policy directions aimed at enhancing the health benefits of green spaces for aging populations.

## 2. Methods

### 2.1. Search Strategy

The search strategy was designed around three main categories: green space, older adults, and health. The Boolean string applied is as follows: “(‘green space’ OR ‘urban green∗’ OR ‘nature exposure’ OR ‘natural environment∗’ OR ‘parks’ OR ‘greenspace’ OR ‘vegetation cover’ OR ‘tree cover’ OR ‘gardening’ OR ‘green infrastructure’) AND (‘older adults’ OR ‘elderly’ OR ‘older people’ OR ‘seniors’ OR ‘aging population’ OR ‘older individuals’ OR ‘geriatric’ OR ‘65 years’ OR ‘senior citizens’) AND (‘health’ OR ‘well‐being’ OR ‘quality of life’ OR ‘mental health’ OR ‘physical health’ OR ‘cognitive function’ OR ‘physical activity’ OR ‘social interaction’).”

This strategy was applied in the Web of Science Core Collection (WoSCC) using the Topic (TS) field and in Scopus using the TITLE‐ABS‐KEY field, thus covering titles, abstracts, and keywords in both databases. To ensure relevance, the search was limited to peer‐reviewed articles published in English between 2004 and 2024, and only studies involving human populations were considered.

### 2.2. Screening and Data Extraction

**Figure 1:**
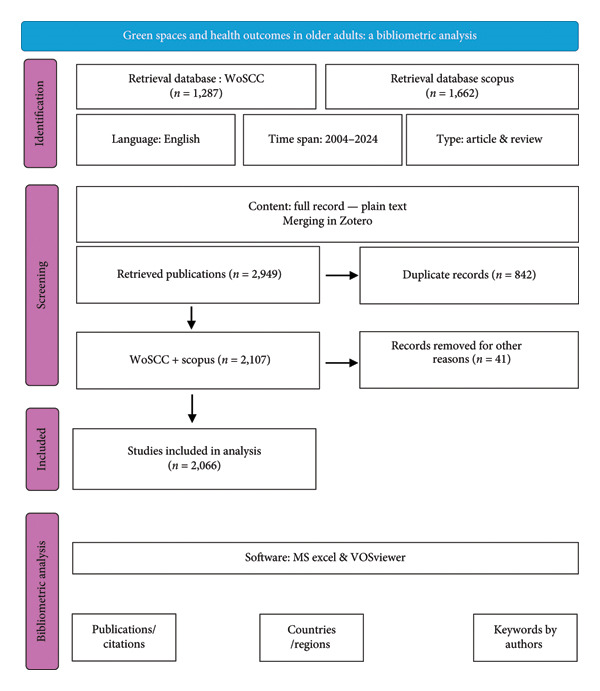
Flow diagram of the data collection.

The data were collected on December 14, 2024. I applied filters to include only English‐language articles and review articles published between 2004 and 2024. The selection of 2004 as the commencement year establishes a 20‐year analytical period, facilitating the observation of evolving research trajectories. This time frame is strategically chosen to encompass the impact of pivotal policy initiatives, notably the World Health Organization (WHO)’s Active Aging: A Policy Framework and the Global Age‐Friendly Cities Guide, which significantly underscored the importance of physical activity, aging, and urban planning in public health discourse [6, 7]. Both datasets were transferred to the reference management software Zotero for organization and merging. To classify and visualize the datasets, VOSviewer and MS Excel were used. The PRISMA 2020 flow diagram detailing the article selection process is presented in Figure 1.

Certain analyses—specifically the cluster map of keywords by author, the geographic distribution of articles, and the identification of the top 20 countries with the most publications—were conducted exclusively using the WoSCC dataset, given its established coverage and citation indexing features. In contrast, both Scopus and WoSCC were employed for the analysis of the number of annual publications and citations, as well as for identifying the top 20 most cited articles, to enhance comprehensiveness and reduce database bias. To supplement our analysis, we performed a rapid screening of article titles for specific keywords (e.g., “cohort,” “longitudinal,” “prospective,” “trial,” “RCT,” and “intervention”). This approach served as a proxy for estimating the proportion of longitudinal and experimental studies within the literature. It is important to note that this method likely underestimates the true prevalence, as it cannot identify studies that do not explicitly declare their design in the title.

## 3. Results

**Figure 2:**
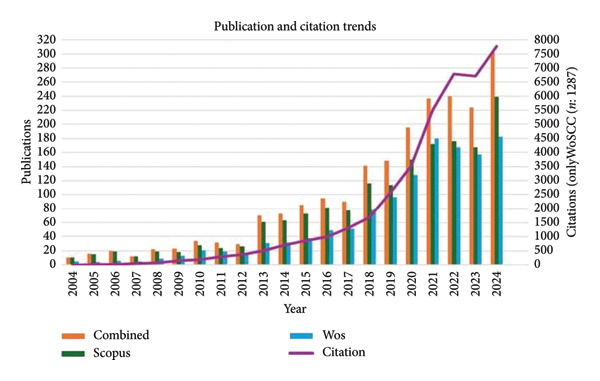
Number of annual publications and citations.

The combined search of WoSCC and Scopus databases yielded a corpus of 2066 publications examining the relationship between green spaces and health in older adults. The articles from WoSCC, which constitute a major part of this collection, have garnered a total of 38,070 citations, with an average of 29.72 citations per article. The publication and citation trends are illustrated in Figure 2. Within this body of literature, a keyword screening of titles and abstracts identified 387 studies (18.7%) as employing longitudinal, cohort, or experimental designs. It is noteworthy that 258 of these (66.7%) were published between 2020 and 2024, a finding that signals a rapid and recent adoption of more rigorous methodological frameworks within the discipline.

**Figure 3:**
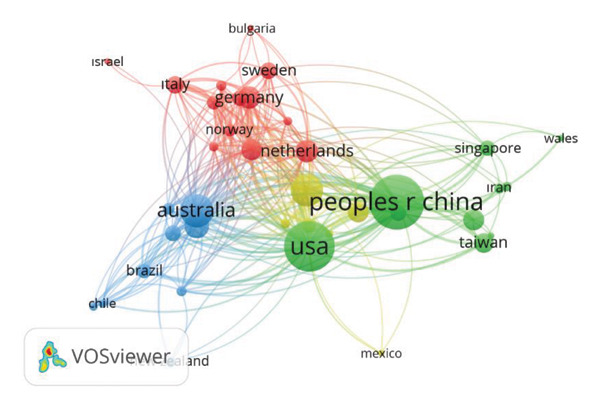
Geographic distribution of origin of articles (WoSCC).

Figure 3 and Table 1 show the geographical distribution of countries with at least five articles and the cocitation network related to the study of green spaces and the health of older adults. The most productive countries are China (PRC) (*n* = 388), the United States of America (USA) (*n* = 329), and Australia (*n* = 142). Among countries, those with the highest citation counts are the United States (12015 citations), PRC (7939 citations), and England (6504 citations).

**Table 1:** Top 20 countries with the most publications (WoSCC).

Rank	Country	Number of publications	Number of citations	First publication year	Publication per year (from first publication year)	Year with the highest publication number
1	PRC	388	7939	2010	27.71	2024
2	United States	329	12,015	2004	16.45	2021
3	Australia	142	4129	2006	7.89	2024
4	England	136	6504	2004	6.80	2024
5	Canada	78	3497	2008	4.88	2021
6	Netherlands	65	3901	2006	3.61	2019
7	Germany	63	2409	2010	4.50	2021
8	Japan	56	1000	2004	2.80	2024
9	South Korea	55	869	2011	4.23	2024
10	Spain	52	1878	2013	4.73	2024, 2020
11	Taiwan	47	935	2010	3.36	2020
12	Italy	38	1098	2005	2.00	2020
13	Sweden	34	1650	2004	1.70	2021
14	Belgium	33	1417	2013	3.00	2024
15	Scotland	33	1478	2011	2.54	2021
16	Poland	28	699	2015	3.11	2022
17	Singapore	28	759	2017	4.00	2020, 2022
18	Brazil	26	660	2010	1.86	2022, 2023
19	France	26	995	2004	1.30	2021
20	Finland	24	654	2008	1.50	2018, 2019, 2020, 2021, 2024

**Figure 4:**
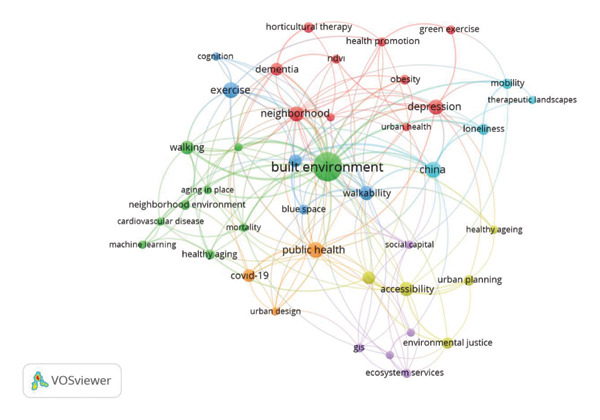
Cluster map of keywords by author on Web of Science Core Collection (WoSCC).

To analyze the trends related to green spaces and older people’s health, based on co‐occurrence, an author keyword cluster map was produced by VOSviewer in Figure 4. Those keywords gathered into seven clusters as Cluster 1 with 10 items (dementia, depression, green exercise, health promotion, horticultural therapy, normalized difference vegetation index [NDVI], neighborhood, obesity, self‐related health, and urban health), Cluster 2 with nine items (aging in place, built environment, cardiovascular disease, healthy aging, machine learning, mortality, neighborhood environment, social environment, and walking); Cluster 3 with five items (air pollution, blue space, cognition, exercise, and walkability); Cluster 4 with five items (accessibility, environmental justice, healthy aging, urban parks, and urban planning); Cluster 5 with five items (climate change, ecosystem services, GIS, social capital, and vulnerability); Cluster 6 with four items (China, loneliness, mobility, and therapeutic landscapes); and Cluster 7 with three items (COVID‐19, public health, and urban design). The most frequent author keywords are built environment (*n* = 123), China (*n* = 38), exercise (*n* = 37), public health (*n* = 37), and depression (*n* = 35).

Table 2 shows the most cited top 20 article lists. The most cited article is “Green space, urbanity, and health: how strong is the relation?” with a total of 1254 WoSCC and 1438 Scopus citations. The article with the highest average of 83.5 citations per year is “Spending at least 120 minutes a week in nature is associated with good health and wellbeing.”

**Table 2:** Top 20 cited articles.

Rank	Title	Authors	Source title	Publication year	WoSCC		Scopus
					Total citations	Average per year	Total citation
1	Green space, urbanity, and health: how strong is the relation?	Maas, J. et al.	Journal of Epidemiology and Community Health	2006	1254	66.0	1438
2	Spending at least 120 minutes a week in nature is associated with good health and wellbeing	White, M. et al.	Scientific Reports	2019	501	83.5	611
3	Built environmental correlates of older adults’ total physical activity and walking: a systematic review and meta‐analysis	Barnett, D. et al.	International Journal of Behavioral Nutrition and Physical Activity	2017	510	63.8	552
4	The relationship between social cohesion and urban green space: an avenue for health promotion	Jennings, V.; Bamkole, O.	International Journal of Environmental Research and Public Health	2019	439	73.2	530
5	Greenspace and obesity: a systematic review of the evidence	Lachowycz, K.; Jones, A. P.	Obesity Reviews	2011	429	30.6	468
6	Green spaces and general health: roles of mental health status, social support, and physical activity	Dadvand, P. et al.	Environment International	2016	380	42.2	417
7	‘Cultivating health’: therapeutic landscapes and older people in northern England	Milligan, C.; Gatrell, A.; Bingley, A.	Social Science & Medicine	2004	351	16.7	413
8	Using deep learning to examine street view green and blue spaces and their associations with geriatric depression in Beijing, China	Helbich, M. et al.	Environment International	2019	357	59.5	393
9	Neighborhood greenspace and health in a large urban center	Omid K. et al.	Scientific Reports	2015	295	29.5	335
10	Neighborhood effects on heat deaths: social and environmental predictors of vulnerability in Maricopa County, Arizona	Harlan, Sharon L. et al.	Environmental Health Perspectives	2013	316	26.3	333
11	Therapeutic landscapes and wellbeing in later life: impacts of blue and green spaces for older adults	Finlay, J. et al.	Health & Place	2015	291	29.1	329
12	The impact of the physical and urban environment on mental well‐being	Guite, H. F.; Clark, C.; Ackrill, G.	Public Health	2006	262	13.8	319
13	Interactions between psychosocial and built environment factors in explaining older adults’ physical activity	Carlson, J. et al.	Preventive Medicine	2012	291	22.4	316
14	The effects of naturalness, gender, and age on how urban green space is perceived and used	Sang, A. O. et al.	Urban Forestry & Urban Greening	2016	278	30.9	313
15	Respiratory and cardiovascular responses to walking down a traffic‐polluted road compared with walking in a traffic‐free area in participants aged 60 years and older with chronic lung or heart disease and age‐matched healthy controls: a randomized, crossover study	Sinharay, R. et al.	The Lancet	2018	277	39.6	312
16	The association between green space and mental health varies across the lifecourse. A longitudinal study	Astell‐Burt, T.; Mitchell, R.; Hartig, T.	Journal of Epidemiology and Community Health	2014	269	24.45	299
17	Neighborhood environments and socioeconomic inequalities in mental well‐being	Mitchell, R. J. et al.	American Journal of Preventive Medicine	2015	203	20.3	284
18	The health benefits of nature‐based solutions to urbanization challenges for children and the elderly – a systematic review	Kabisch, N.; van den Bosch, M.; Lafortezza, R.	Environmental Research	2017	229	28.6	264
19	Dose‐response relationship between physical activity and mental health: the Scottish Health Survey	Hamer, M.; Stamatakis, E.; Steptoe, A.	British Journal of Sports Medicine	2009	235	14.7	256
20	Urban green spaces for the social interaction, health and well‐being of older people – an integrated view of urban ecosystem services and socio‐environmental justice	Enssle, F.; Kabisch, N.	Environmental Science & Policy	2020	226	45.2	252

## 4. Discussion

Our findings demonstrate a consistent increase in publications on green spaces and older people’s health from 2004 to 2024, with notable surges in 2014 and 2018. This trend is fundamentally driven by the global priority shift toward aging populations, which propelled concepts such as the WHO’s “age‐friendly cities” to the forefront of public health and urban policy agendas [6, 7]. The initial surge in 2014 can be attributed to a confluence of factors, including the maturation of the research field and its specific application to aging demographics. This was powerfully catalyzed by the WHO’s 2013 Global Action Plan for the Prevention and Control of Noncommunicable Diseases, which emphasized creating recreational spaces to reduce inactivity, thereby aligning closely with the core principles of age‐friendly cities and this research field [8].

The second surge in 2018 was arguably driven by the overarching policy framework of the United Nations’ Sustainable Development Goals (SDGs), which explicitly linked urban sustainability with health and well‐being for all ages, further stimulating interdisciplinary research [9]. Despite a postpandemic slowdown in the rate of increase, the highest number of publications was recorded in 2024, underscoring the enduring and growing significance of this field of study.

A recent meta‐analysis revealed that green space exposure is associated with reduced risks of depression and anxiety, with stronger protective effects observed among women and rural populations [1]. The health benefits of contact with natural environments operate through multiple biopsychosocial pathways, such as harm reduction (e.g., lowered exposure to air pollution, noise, and extreme heat), restoration (e.g., recovery of attentional capacity and stress reduction), and capacity building (e.g., encouraging physical activity and strengthening social cohesion). For older adults, proximity to green spaces is particularly important due to mobility limitations. Studies show that higher levels of neighborhood greenness are associated with improved mental health outcomes, and these effects tend to be strongest among socioeconomically disadvantaged groups. Promoting mental health in this way is a key factor in fostering healthy, longer lives, underscoring the role of green spaces in supporting both immediate well‐being and long‐term resilience in older populations [5]. From a broader socioecological perspective, in addition, longevity is also profoundly shaped by structural determinants such as health expenditures, social support systems, and societal inequality. As Kim notes, these contextual factors substantially influence the probability of surviving to very old age, suggesting that the mental health benefits of green spaces may be most impactful when complemented by supportive policy and community infrastructures [10].

The analysis of publication metrics reveals distinct patterns of contribution and influence in this research field. China, the United States, and Australia emerged as the top three producers of scientific output, with 388, 329, and 142 publications, respectively, a finding reinforced by their high average annual publication rate since their first contributions (Table 1). This leadership in volume is further reflected in citation counts, where the United States leads with 12,015 total citations, followed by China (7939) and England (6504), underscoring the significant impact of their work. The early engagement of the United States and Australia since 2004 has laid the foundation for their sustained output and influence. Australia, in particular, has maintained a strong presence, with a notable citation impact (4129) supported by robust health promotion initiatives and urban green space policies [11]. When adjusted for population size, a different pattern of contributions emerges. Looking at publications on a per capita basis highlights which nations devote proportionally greater attention to this topic. By this metric, Switzerland (2671), Denmark (2630), and Norway (2465) stand out as the leading countries. Of the top three in overall output, only Australia remains highly ranked, placing fourth (2372), whereas China and the United States do not appear in the top 20 [12]. This contrast indicates that although larger countries dominate in absolute numbers of publications, the issue carries relatively greater national priority in smaller, wealthier nations, where it is more closely tied to public health and age‐friendly urban planning agendas.

Interestingly, all top 10 contributing countries have old‐age dependency ratios higher than the global average of 15.5%, ranging from 20.7% in China to 50.1% in Japan. More than half of these nations report median ages over the 40s, indicating aging populations that likely drive research interest in green spaces as a strategy to enhance older adults’ health and quality of life. Countries such as Japan, where 28% of the population is aged 65 or older, exemplify the intersection of aging demographics and the growing need for evidence‐based interventions such as green spaces to support healthy aging [13]. This demographic pressure likely functions as a key driver, creating a pragmatic demand for evidence‐based solutions, such as urban greening, to alleviate the public health burdens associated with an aging society.

However, high productivity does not necessarily translate into equitable access or consistent quality. The availability and benefits of green spaces are far from uniform, even among the countries leading this field of research. Although nations in Northern and Western Europe often boast higher urban green space coverage, their Southern and Eastern counterparts tend to lag behind [14]. Furthermore, consistent and up‐to‐date data are scarce, especially in rapidly developing nations such as China, making it difficult to get a true global picture. Crucially, the WHO warns that simply having green space is not enough; equitable access is a persistent challenge. They note that parks in lower‐income or minority neighborhoods are often of poorer quality, less safe, and poorly maintained, meaning the health benefits documented in studies may not extend equally to all older adults [15]. This idea is echoed by Jennings and Bamkole, who point out that the design and condition of a space are paramount. A park that is rundown or feels unsafe can hinder social interaction instead of promoting it. For older adults, certain features such as dense tree cover or very large parks can even be perceived as safety hazards [16]. This pattern was also observed in Berlin, where research by Kabisch and Haase found that older residents and those in immigrant‐dense inner‐city neighborhoods used large parks like Tempelhof less frequently, citing a lack of age‐friendly infrastructure [17]. Adding to this perspective, a recent study shows that even simulated exposure can yield benefits: For instance, an immersive virtual reality study demonstrated reductions in symptoms of depression and stress, alongside improvements in happiness and relaxation among older participants [18]. Together, these insights shift the focus from merely quantifying green space to qualifying it, emphasizing that its health benefits for aging populations depend entirely on thoughtful design, dedicated maintenance, and truly inclusive access.

While these benefits are increasingly recognized, ensuring equitable access remains a major challenge. The equitable distribution of benefits of green space is often undermined by a process known as “environmental gentrification.” In this situation, well‐intentioned investments in parks and greenery can lead to rising property values and living costs, which may force out vulnerable groups—such as older, long‐term residents—who were originally intended to benefit from these improvements. This creates a contradictory outcome where public health and urban greening efforts contribute to socioeconomic displacement. To avoid this, it is critical to involve older residents directly in the planning process and to adopt complementary housing measures that prevent displacement, ensuring that green infrastructure serves everyone equitably [19]. In this context, national policy environments also play a role. For example, England, Germany, and the Netherlands are among the top contributors, with England standing out for its high citation count (6504) and steady publication trends. The Netherlands, while producing fewer publications, demonstrates a strong citation‐to‐publication ratio, suggesting a high influence of its research contributions. Countries such as Australia and the Netherlands, which prioritize green space in urban planning, may benefit from supportive policies that facilitate research on the health impacts of green environments.

Beyond country‐level and policy contributions, thematic analysis further illustrates how research priorities have evolved. Keyword analysis revealed seven thematic clusters, underscoring the highly interdisciplinary character of this field. Cluster 1, for instance, revolved around mental health and psychological well‐being, featuring prominent terms such as “dementia,” “depression,” and “green exercise.” This suggests a dominant research focus on the role of natural environments in mitigating cognitive decline and improving mental health. The high frequency of terms such as “built environment” (*n* = 123), “exercise” (*n* = 37), and “depression” (*n* = 35) further points to a robust research interest in how urban design and physical activity interact to influence mental health, marking these themes as priority areas for further study. Overall, green spaces are increasingly perceived as a versatile public health intervention that addresses a spectrum of needs in aging populations. Beyond their established role in promoting mental health and physical activity, contemporary studies, such as those by Jennings and Bamkole, increasingly highlight their significance in fostering social cohesion and building social capital, which are vital for psychological well‐being [16]. Nonetheless, these authors call for more research into how specific types and qualitative features of green spaces shape these social benefits. Integrating social cohesion into theoretical models that link green space to health could yield a more holistic understanding, which is especially relevant for older adults who are at greater risk of social isolation [16].

Some countries with significant aging populations, such as Japan and South Korea, demonstrate moderate research productivity in this area (65 and 55 publications, respectively), suggesting potential for further research expansion. Temporal trends also reveal peaks in publication numbers in 2024 for many countries, reflecting heightened global interest in nature‐based solutions for health and well‐being. Notably, nations such as Finland, Brazil, and Singapore have shown recent increases in research output, signaling growing recognition of the role of green spaces in public health.

Our analysis reveals that longitudinal and experimental studies currently represent a relatively small proportion (18.7%) of the literature on green spaces and older adult health. However, a notable upward trend is emerging, with two‐thirds of these more methodologically robust studies having been published in the last 5 years. This suggests a pronounced and recent shift toward research designs that are better suited to establish causality and determine long‐term health impacts. Despite this progress, the evidence base remains dominated by cross‐sectional data. Addressing this methodological gap by promoting longitudinal and intervention‐based research is essential to produce higher‐quality evidence that can effectively inform public health policy and urban planning. This evolution is critical because cross‐sectional designs cannot fully capture the complex, bidirectional nature of the relationship between green space exposure and health outcomes. As emphasized by Browning and Rigolon, the field is increasingly focused on moving beyond demonstrating associations to uncovering causal mechanisms and dose–response relationships. They further note that future research should leverage natural experiments and longitudinal data on environmental changes, thereby providing a more comprehensive and powerful understanding of causality [20].

A key strength of this research lies in its broad approach, which investigates the full range of health outcomes linked to green space exposure among older adults, not only mental health or quality of life. This wide‐ranging perspective supports a more holistic view of how natural environments affect aging populations.

Although both WoSCC and Scopus were utilized in this study, some analyses—specifically the cluster map of keywords by author, the geographic distribution of articles, and the identification of the top 20 countries with the most publications—relied solely on WoSCC. Consequently, relevant publications indexed only in Scopus may have been excluded, potentially influencing these findings. Additional limitations include the inclusion of only English‐language articles, which may limit the global representation of findings. Furthermore, although efforts were made to refine the search, some publications might have included additional age groups beyond older adults or referred to older populations only as an exclusion criterion rather than as a central focus of the research. Finally, the identification of longitudinal and intervention‐based studies through rapid title screening may have underestimated their true prevalence, as some studies do not explicitly report their design in titles.

Overall, the emphasis on integrating green spaces into urban planning, particularly in countries with aging populations, underscores the recognized importance of accessible natural environments in promoting the health and quality of life of older adults. While systematic reviews exist on specific subtopics such as mental health, quality of life, and cardiovascular diseases, further reviews are needed to explore a broader range of health outcomes or other subthemes related to older adults’ health outcomes.

## 5. Conclusion

This bibliometric analysis reveals a marked increase in research examining the relationship between green spaces and older adults’ health, with the most substantial growth occurring in the past decade. Part of this rise reflects broader global policy priorities, such as the WHO’s age‐friendly city agenda and the UN SDGs, but it also mirrors the general expansion of scientific publishing and the growing number of researchers in the field. Despite this progress, the evidence base remains dominated by cross‐sectional studies, with relatively fewer longitudinal or intervention‐based designs that could clarify causal mechanisms and long‐term impacts. Expanding research efforts to underrepresented regions and fostering cross‐disciplinary collaborations would help build a more comprehensive and inclusive evidence base. From a policy perspective, the literature emphasizes that beyond increasing the quantity of green infrastructure, ensuring equitable access and mitigating risks such as environmental gentrification are essential to maximizing the benefits of green spaces for aging populations.

## Ethics Statement

The author has nothing to report.

## Disclosure

An earlier version of this work was presented as an oral presentation at the “4th International City, Environment and Health Congress” in 2025.

## Conflicts of Interest

The author declares no conflicts of interest.

## Funding

The author did not receive funding from any organization for the submitted work.

## Data Availability

The data that support the findings of this study are available from the corresponding author upon reasonable request.
